# An amateur gut microbial configuration formed in giant panda for striving to digest cellulose in bamboo: Systematic evidence from intestinal digestive enzymes, functional genes and microbial structures

**DOI:** 10.3389/fmicb.2022.926515

**Published:** 2022-07-26

**Authors:** Mingye Zhan, Aishan Wang, Yong Yao, Yingmin Zhou, Shu Zhang, Xiaohua Fu, Jun Zhou, Enle Pei, Lei Wang

**Affiliations:** ^1^College of Environmental Science and Engineering, Institute of Pollution Control and Ecological Safety, Tongji University, Shanghai, China; ^2^Shanghai Zoo, Shanghai, China; ^3^Chongqing Zoo, Chongqing, China; ^4^China Conservation and Research Center for the Giant Panda, Dujiangyan, China

**Keywords:** giant panda, gut microbiome, cellulose digestion, cellulases activity, dietary adaptation

## Abstract

The giant panda has been considered to maximize nutritional intake including protein and soluble carbohydrates in bamboo, but it has spent almost entire life with the high-cellulose diet. Whether giant panda is still helpless about digesting bamboo cellulose or not is always contentious among many researchers around the world. The work has systematically clarified this issue from the perspectives of digestive enzymes, functional genes, and microbial structures in giant panda gut. The intestinal cellulase activities of panda increase with bamboo consumption, performing that the endoglucanase activity of adults reaches 10-fold that of pandas first consuming bamboo. More abundance and types of microbial endoglucanase genes occur in bamboo-diet giant panda gut, and the corresponding GH5 gene cluster is still efficiently transcribed. Gut microbes possessing cellulose-degrading genes, belong to the phylum Firmicutes and some Bacteroidetes, but their structural and functional configurations are insufficient to completely degrade cellulose. Therefore, giant panda is striving to digest cellulose in bamboo, but this adaptation is incomplete. This is probably related to the short straight carnivore-like gut structure of the giant panda, preventing the colonization of some efficient functional but anaerobic-preferred flora.

## Introduction

Since the 1960s, protection of the giant panda has been undertaken and gradually expanded in China, especially after the construction of the Giant Panda National Park in 2017. There has been marked improvement in the breeding capacity, recovery of severely fragmented habitats, and a reduction in the high inbreeding risk of the population (Kang and Li, 2019; Song et al., 2021). However, the vulnerability of the giant panda remains a global concern, because they have an exclusively bamboo diet despite a carnivore-like intestine and a very poor ability to digest bamboo.

Unlike herbivorous mammals, which have rumens to ferment cellulose, the giant panda relies solely on the microbial flora in its hindgut (Li et al., 2010; Wei et al., 2019). When researchers have studied the gut microbiome of the giant panda after dietary shift, they have detected no colonization by large cellulose-degrading microbial populations in this specialized species (Zhan et al., 2020). However, in a study that analyzed the fecal microbial flora of bamboo-consuming animals, including the adult giant panda and two other species, McKenney et al. (2018) have shown that all three species shared some specialized flora during their long-term adaptation. An association analysis has identified a group of bacteria in the giant panda related to the genus *Clostridium* as candidates for the degradation of cellulose (Zhu et al., 2018; Guo et al., 2020), but their production of the appropriate digestive enzymes has not yet been demonstrated. The data are still insufficient to explain that this microbial flora assists in the giant panda‘s digestion of cellulose, but reflect that the gut flora may possess an adaptive process to the bamboo diet of giant panda.

Zhan et al. (2020) have investigated the ability of the gut microbial flora to digest cellulose by analyzing the microbial genome of the giant panda during its dietary shift during weaning, and found a succession of glycoside hydrolase genes appropriate for cellulose degradation. However, Zhang et al. (2018) have conducted a similar investigation according to different sequencing and analysis methods, but proposed that the giant panda might live on the hemicellulose in bamboo, rather than on the cellulose. Metagenomic analyses of the adaptive changes that allow giant panda to digest bamboo have produced different results in various studies. To clarify the actual ability of the gut flora of the panda to digest cellulose, there is still a need for efficient sequencing technologies with larger data volume and higher sequence quality, combined with relevant research into the transcription of microbial cellulose-degrading genes in the giant panda.

Some studies have also reported the apparent digestibility of cellulose in the adult giant panda (Wang et al., 2006; Lin et al., 2020) or compared the activities of cellulases secreted by the gut flora with those of other herbivores (Guo et al., 2018). However, to fully understand whether the adaptation to a high-cellulose diet occurs in the giant panda, how the panda’s ability to digest cellulose changes during its growth, and what determines its ability (or incomplete ability) to digest bamboo, a systematic study that combines analyses of enzymes, gut microbes, and the abundances and expression of functional genes in its different developmental stages is necessary.

The dietary shift is a notable stage in the giant panda‘s development, occurring between 8 months and 2.5 years of age, and the death rate of the giant panda is very high in this period, mainly with symptoms of gastrointestinal diseases (Zhou et al., 2021). Several studies have identified some defects including reduced microbial diversity and the lack of a dominant cellulose-degrading flora or genes encoding cellulases, in the giant panda during the dietary shift compared with those in the rumen of cattle (Suen et al., 2010; Guo et al., 2020; Jin et al., 2020; Zhan et al., 2020). The systematic analysis from the enzymes, gut microbes and functional genes can comprehensively determine whether it is the most vulnerable stage, during the pandas’ developmental process due to their inability to digest cellulose.

In this study, several pandas at different stages of their development (including cubs and during the dietary shift process, sub-adult and adult, respectively) have been selected for sample collection to: (1) analyze the changes in cellulase activities during the dietary shift and growth periods, reflecting adaption to a high-cellulose diet; (2) profile microbial cellulose-degrading genes by advanced multi-omics technologies, exploring the molecular adaptive response of cellulose digestion; and (3) identify the cellulose-degrading contributors, paralleling the panda’s adaptive change to digesting cellulose. The results may systematically and hierarchically explain the current adaptive development of giant panda for the digestion of bamboo (also including wild giant panda, because they also experience dietary transition from milk to bamboo).

## Materials and methods

### Sample collection

Fecal samples were collected from individual captive giant pandas at different developmental stages (*n* = 22) at the China Conservation and Research Center (Dujiangyan, China), Chongqing Zoo (Chongqin, China), and Shanghai Zoo (Shanghai, China). Detailed information on these individuals is given in Supplementary Table 1. All samples were immediately stored at 0–4°C before enzyme assays and at <−20°C before DNA extraction, and were frozen in liquid nitrogen before the extraction of total RNA. The age and dietary composition of the giant pandas at different developmental stages are shown in Table 1. The health status of the pandas was monitored during the sampling period and no abnormalities were detected.

**TABLE 1:** Feeding and dietary information of giant panda at different developmental stages.

Developmental stage	Age	Dietary composition
**Cub**	**0∼1 year**	**Milk-fed** (mainly ate milk, licking a little coarse pastry and bamboo shoots)
**The dietary shift stage**	**1∼2 years**	**Dietary shift** (milk consumption decreased and bamboo consumption increased, with bamboo gradually excreted in the feces)
**Sub-adult**	**>2∼6 years**	**Bamboo-fed** (mainly ate bamboo, supplemented with little bamboo shoots, coarse pastry, apple and carrot)
**Adult**	**>6 years**	

### Enzyme activity assays

A 5 g sample of giant panda stool was stored at 4°C. It was then placed in 50 mL of citrate buffer solution (pH 4.8), mixed thoroughly, and centrifuged at 4000 rpm (10 min) to obtain the crude cellulase solution. Carboxymethyl cellulose solution (1%, 10 mL) was added to 5 mL of enzyme solution. The mixture was stored at 40°C for 30 min before the endoglucanase activity was measured at 540 nm with the 3,5-dinitrosalicylic acid (DNS) colorimetry method (Ghose, 1987). Microcrystalline cellulose (100 mg) was mixed with 10 mL of satellite salt buffer solution (pH 4.8), and then reacted with 5 mL of the above enzyme solution for 1 day at 40°C. The exoglucanase activity was measured at 540 nm with the DNS method (Han and Chen, 2010; Alawlaqi and Alharbi, 2020). The activity of crude protease was measured with the Folin method at pH 7.5 (SB/T 10317-1999) (SB/T 10317-1999, 1999), and the amylase activity was analyzed by measuring the production of reducing sugar from soluble starch with DNS (Stroparo et al., 2012). We set up parallel experiments (*n* = 3), using a no-substrate control (in which distilled water replaced the substrate solution).

### DNA extraction and sequencing

The frozen stool samples from the giant pandas were thawed on ice packs. A contamination-free core part of each sample (10 g/day) was taken from five stool samples collected on five consecutive days with a clean, sterile pharmacy spoon. The samples from each individual were mixed for later analysis. The DNA was extracted from the fecal samples with the PowerSoil^®^ DNA Isolation Kit (Mo Bio Laboratories, Inc., Carlsbad, CA, United States). The extracted DNA was stored at <−20°C before analysis. The details of the DNA extraction, and 16S rRNA and internal transcribed spacer 1 (ITS-1) sequence PCR amplification are available in the Supplementary material. The raw reads were deposited in the National Center for Biotechnology Information (NCBI) Sequence Read Archive (SRA) database.

### 16S rRNA, internal transcribed spacer 1, and metagenomic sequence analyses

A pyrosequencing analysis was performed on the Illumina MiSeq PE300 platform by Shanghai Majorbio Technology Co., Ltd. (Shanghai, China). The raw fastq files for the 16S rRNA and ITS rRNA were demultiplexed and quality-filtered with fastp version 0.19.6 (Chen et al., 2018) and merged with FLASH (version 1.2.11) (Magoč and Salzberg, 2011). The operational taxonomic units (OTUs) were clustered with a 97% similarity cutoff using UPARSE (version 7.1) (Edgar et al., 2011). Chimeric sequences were identified and removed with UCHIME. The taxonomy of representative sequences of each OTU was analyzed with RDP Classifier version 2.11 (Wang et al., 2007) against the 16S rRNA (Silva version 132) and ITS (UNITE version 8.0) databases with a confidence threshold of 0.7.

The concentration and purity of the extracted fecal DNA (samples from before, during, and after the dietary shift; *n* = 6 total) were determined with a TBS-380 Mini-Fluorometer (Turner BioSystems, San Francisco, CA, United States) and a NanoDrop 2000 spectrophotometer (NanoDrop, Wilmington, DE, United States), respectively. A paired-end library was constructed with the NEXTFLEX™ Rapid DNA-Seq Kit (Bioo Scientific, Austin, TX, United States). Paired-end sequencing was performed on the Illumina NovaSeq platform (Illumina Inc., San Diego, CA, United States) at Majorbio Bio-Pharm Technology Co., Ltd. (Shanghai, China) using the NovaSeq Reagent Kit. The paired-end Illumina reads were qualified with Transcriptomic Sequence Analysis Fastp (version 0.20.0) (Chen et al., 2018). The metagenomic data were assembled with MEGAHIT (version 1.1.2) (Li et al., 2015). The open reading frames in each assembled contig were predicted with MetaGene (Noguchi et al., 2006). The predicted ORFs (≥100 bp) were retrieved and translated (by NCBI). A non-redundant gene catalog was constructed with CD-HIT (version 4.6.1) with 90% sequence identity and 90% coverage (Fu et al., 2012). After quality control, the remaining reads were mapped to the gene sets with SOAPaligner (version 2.21) with 95% identity (Li et al., 2008), and the gene abundance in each sample was evaluated. Representative sequences from the NR gene catalog were aligned to those in different functional databases for taxonomic annotation (following best-hit approach) and KEGG annotation by DIAMOND (version 0.8.35) at an optimized e-value cutoff of 1e–5, and carbohydrate-active enzyme annotation *via* HMMER (version 3.1. b2) with an e-value cutoff of 1e–5 (Buchfink et al., 2015). The criteria for DNA quality and 16S, ITS-1, and metagenomic sequencing are available in the Supplementary material, together with details of the construction of the paired-end library and the annotation of sequences.

### Transcriptome sequencing analysis

The total RNA was extracted from the fresh giant panda fecal samples with the RNA PowerSoil^®^ Total RNA Isolation Kit (Mo Bio Laboratories Inc.) and immediately stored in liquid nitrogen. The rRNA was removed with the magnetic bead method to isolate the mRNA. A cDNA library was constructed with the TruSeq Stranded mRNA LT Sample Prep Kit (Illumina Inc.). The reads were qualified on an Agilent 2100 Bioanalyzer (Agilent Technologies Inc., Beijing, China) with the Agilent High Sensitivity DNA Kit, and quantified on the Promega QuantiFluormeter (Promega Corporation, Beijing, China) with the Quant iT™ PicoGreen™ dsDNA Assay Kit. Paired-end sequencing was performed on the Illumina NovaSeq platform (Illumina Inc.) at Shanghai Personal Biotechnology Co., Ltd. (Shanghai, China) using the NovaSeq 6000 S4 Reagent Kit (2 bp × 150 bp). The raw reads were deposited in the NCBI Sequence Read Archive (SRA) database. The details of the criteria for RNA quality and the construction of the paired-end library and annotation of sequences are available in the Supplementary material.

### Statistical analysis

All data on digestive enzyme activities, the abundances of genes and transcripts, and microbial α-diversity in the panda feces were calculated with OriginPro 8 SR0 (version 8.0724). Significant differences in α-diversity between groups were detected with Welch’s *t*-test. The R language vegan package was used to draw the OTU community bar charts and heatmaps. The Bray–Curtis method was used to calculate the distance between two samples and to quantify the differences in the species abundance distributions among samples. The OTU abundance table was standardized with Phylogenetic Investigation of Communities by Reconstruction of Unobserved States 2 (PICRUSt 2), a software package for the functional prediction of amplified 16S rRNA sequences, which removes the effect of the copy number of the 16S rRNA gene in the species genome. Each OTU corresponding to a phylogenetic lineage in this software was then used to annotate the Clusters of Orthologous Genes and KEGG functions of the OTUs to produce the OTU annotation information for each functional level and the abundance information for each function. PICRUSt 2 was also used to analyze the fungal data.

## Results

### Enzyme activities and properties related to bamboo digestion in giant panda at different developmental stages

**FIGURE 1:**
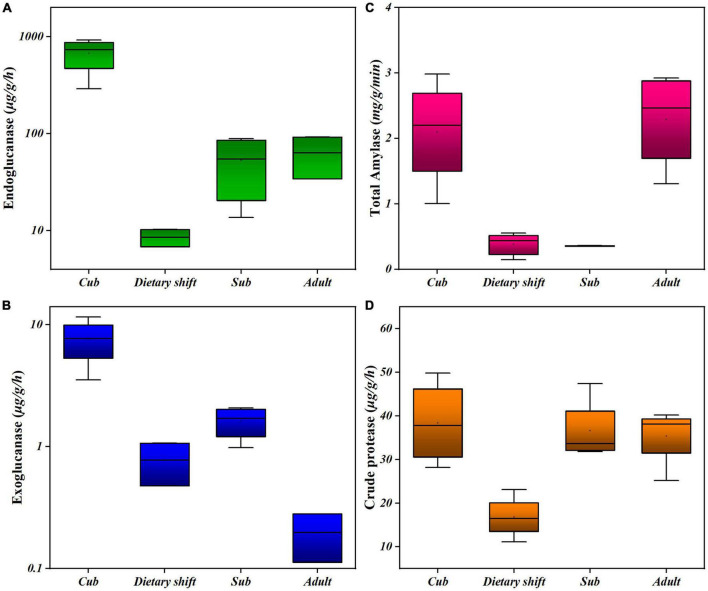
The activities of different digestive enzymes in giant panda during its development. **(A)** Endoglucanse activity; **(B)** exoglucanase activity; **(C)** total amylase activity; **(D)** crude protease activity. Each box has reflected the range of digestive activities of individual pandas (*n* > 3) at the specific developmental stage.

In considering the adaptation of the giant panda to a diet of bamboo (with a predominant cellulose content) during its development, the activities of endoglucanase and exoglucanase were examined in individual pandas at different developmental stages, using carboxymethyl cellulose and microcrystalline cellulose as substrates, respectively. The enzyme activities were measured as the production of reducing sugar (Figures 1A,B). These enzymes were two key cellulases, hydrolyzing the non-crystalline and crystalline regions of the cellulose molecule, respectively (Attigani et al., 2016). Surprisingly, during the development of the giant panda, both the endoglucanase and exoglucanase activities were initially high in cubs, but decreased during the dietary shift process. They slowly recovered, and thus presented *U*-shaped activity profiles, as did the other digestive enzymes in the feces, e.g., amylase and crude proteases. However, the activities of both cellulases did not recover as well as those of the other enzymes (Figures 1C,D), and the exoglucanase activity was always 1–2 two orders of magnitude lower than the endoglucanase activity (Figure 1).

Changes in all the microbial enzyme activities indicate that the gut flora formed in childhood are under great pressure to digest the high-cellulose diet when the panda first begins to consume bamboo, reflecting the difficulty faced by the giant panda in transition to a bamboo diet. However, giant panda gradually partially digests bamboo cellulose with the assistance of its microbiome.

**FIGURE 2:**
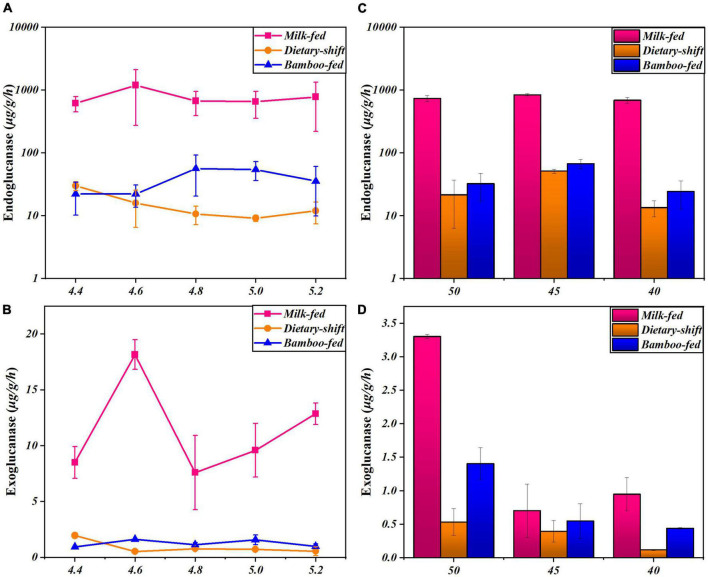
The activities of endoglucanase and exoglucanase in giant panda at different developmental stages under different pH and temperature conditions. **(A,B)** Represented respectively the activities of endoglucanase and exoglucanase of the giant pandas on milk-fed diet, dietary-shift process and bamboo-fed diet, under different pH at 4.4∼5.2 (*T* = 40°C); **(C,D)** represented respectively the activities of endoglucanase and exoglucanase of giant pandas at different dietary stages under different temperature at 40∼50°C (pH 4.8). Each point or column has reflected the range of digestive activities of individuals (*n* > 3) at the specific developmental stage and culture condition.

Cellulase activity was further monitored at different pH and temperature in the giant panda at different developmental stages to explore the changes in the properties and types of microbial cellulases in giant pandas during the adaptation to eating bamboo. The relationship between endoglucanase activity and pH was clearer in the adults on a bamboo diet. Endoglucanase of adults showed the highest activity at pH 5, which was similar to the fecal pH (Figure 2). However, the endoglucanases in the milk-fed cubs did not respond in any way to pH (Figure 2A). This means that the properties and types of endoglucanase in adults might differ from those in cubs, and the endoglucanase in adults may occur to digest cellulose in bamboo.

### Genes encoding lignocellulose-decomposing enzymes in gut microbial genome and transcriptome of giant panda

To explain why the ability of the giant panda to digest bamboo cellulose is still low and why it cannot completely adapt to a bamboo diet, we analyzed the abundances of cellulolytic genes and their transcription in the giant panda at different developmental stages.

**FIGURE 3:**
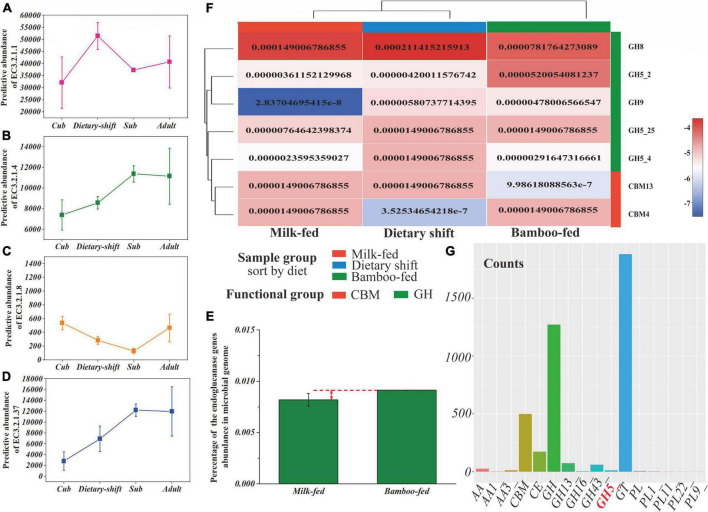
The gene frequency and abundance of cellulase for giant panda during the dietary adaptation process and the transcription efficiency of GHs in gut flora. **(A–D)** Represented the gene frequency of α-amylase (EC 3.2.1.1), endoglucanase (EC 3.2.1.4), endoxylanase (EC 3.2.1.8), and β-D-xylosidase (EC 3.2.1.37), respectively, of the giant panda at cub, dietary-shift, sub-adult and adult stages; **(E)** showed the heat map of GHs genes corresponding to cellulose degradation in the giant panda with different dietary compositions, calculated with the relative abundance in genome; **(F)** represented the percentage of endoglucanase (EC 3.2.1.4) gene abundance in the giant panda on milk-fed diet and bamboo-fed diet, and **(G)** represented the counts of gene transcripts of all classes of carbohydrate-active enzymes and their main families of gut microbiome in the bamboo-fed giant panda.

The data set of non-redundant genes (total value = 724 019) collected from all metagenomic samples was annotated with the Kyoto Encyclopedia of Genes and Genomes (KEGG) database. The metabolic level 2 categories showed that genes related to carbohydrate metabolism (total value = 31 130) were most abundant in the gut microbiome of giant panda, while polysaccharide metabolism accounted for only 21% and even less was related to the degradation of cellulose and hemicellulose. Cellulose and hemicellulose are both more readily degraded than lignin in the lignocellulose complex (Wilhelm et al., 2019; Zhang et al., 2019). Amino acid metabolism (total value = 21 368) and nucleotide metabolism (total value = 12 767) followed. Therefore, we focused on the key enzyme genes involved in the hydrolysis of cellulose and hemicellulose, including endoglucanase (EC 3.2.1.4), endoxylanase (EC 3.2.1.8), and β-D-xylosidase (EC 3.2.1.37) the frequency and relative abundance of which were indeed much less than α-amylase (EC 3.2.1.1) (Figures 3A–D).

Consistent with the results of the enzyme activity analysis, no genes encoding exoglucanase (EC 3.2.1.91) were predicted and had disappeared from the microbial genome. The abundances of key hemicellulose-degrading genes were always 1–2 orders of magnitude lower than those of the cellulose-degrading genes during the development of the giant panda. As shown in Figures 3B,E, the frequencies and relative abundances of endoglucanase (EC 3.2.1.4)-encoding genes increased during the panda’s development and with bamboo consumption (reaching to nearly 0.01%, gene count/total gene count), although the relative abundance of endoglucanase in the adult giant panda was still only one-half that in herbivores (Guo et al., 2018).

Alignment of the above-mentioned NR genes to CAZy database identified 377 different CAZy families and subtypes, including 7134 genes encoding 184 glycoside hydrolases (GHs) and subtypes. Surprisingly, the numbers and types of GHs corresponding to endoglucanase (EC 3.2.1.4) also increased in the giant panda after it adopted a bamboo diet, including the two subtypes of GH5 (GH5_2 and GH5_4) and GH9 (Figure 3F). GH5 is always more abundant during the development of the giant panda (Zhan et al., 2020), with a relative abundance of 0.0056 among the GHs (gene count/total gene count of GHs) (Supplementary Table 2). The genes encoding GH9 were only found after consumption of a bamboo diet, but were not abundant, with a relative abundance of 0.0019 among the GHs (Supplementary Table 2). GH9 has been reported to degrade complex celluloses (Vita et al., 2019).

In an analysis of the transcriptome, GH5, GH13, GH16, and GH43 were the only GHs transcribed in the gut flora of the bamboo-fed giant pandas (Figure 3G), and among these, only GH5 is reported to degrade cellulose, with a relative abundance of 0.0094 among the GH transcripts. No GH9 transcripts were detected. However, the transcripts of GH13 and GH43, which encode proteins responsible for oligosaccharide degradation, were more abundant (Dai et al., 2014).

These results suggested that the relative abundance and type of genes encoding endoglucanase (EC 3.2.1.4) were much greater in the bamboo-fed giant panda, which could endow the giant panda with the ability to digest bamboo cellulose. However, the cellulase activity in the giant panda might still be restricted by the lack of available genes for the hydrolysis of the complex celluloses in bamboo.

### Gut microbial diversity and composition of giant panda at different developmental stage and the microbial contributors for digesting bamboo

The occurrence and accumulation of cellulase genes in the gut microbial genome of the giant panda, together with GH5 transcripts in the gut microbial transcriptome, indicate that the gut flora of the giant panda contributes to its adaptation to a bamboo diet. The changes in the structure and composition of the gut flora were further studied, especially the cellulolytic contributors to the panda’s digestion of cellulose as the cub matures into an adult.

**FIGURE 4:**
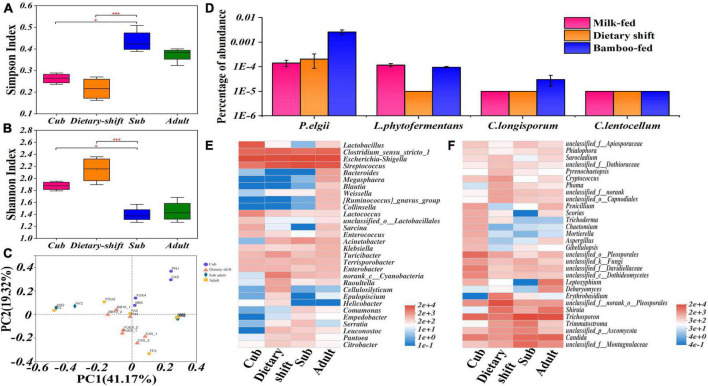
The diversity and structure of gut flora and the relative abundance of microbial populations possessing endoglucanase genes in giant panda at different developmental stages along with the adaptation to bamboo diet. **(A,B)** Represented the α analysis of Simpson’s and Shannon’s Index in growth of the giant panda, respectively, and also have shown the significant difference (0.01 < *P* ≤ 0.05, marked with *; 0.001 < *P* ≤ 0.01, marked with **; *P* ≤ 0.001, marked with ***) of these indexes among different stages; **(C)** β diversity analysis based on Bray-Curtis method has quantified the differences in the species abundance distributions among the samples at different developmental stages; **(D)** has shown the relative abundance of microbial populations possessing endoglucanase (EC 3.2.1.4) genes in the giant panda at different dietary stages; **(E,F)** were heat maps that have shown the succession of bacterial and fungal composition respectively in the giant panda.

In an α-diversity analysis, both Shannon’s and Simpson’s index varied significantly (*P* < 0.05) between the cub and the sub-adult, indicating that the microbial diversity became more complex during the dietary shift process. The Shannon’s index of the gut microbiome then decreased significantly (*P* < 0.05, Figures 4A,B). A β-diversity analysis indicated that the differences in the gut microbial structure among individuals increased after the dietary shift (Figure 4C). It seems that the gut microbes of the giant panda form a single-diversity and sensitive community under the influence of the individual environment, when the giant panda first mainly consumes bamboo.

An investigation of the gut microbial composition showed that the phyla Firmicutes and Proteobacteria were always predominant, with the relative abundance of >90%. Among them, five genera (*Streptococcus*, *Clostridium*, *Megasphaera*, *Blautia*, and *Weissella*) of Firmicutes and three genera (*Escherichia*-*Shigella*, *Acinetobacter*, and *Klebsiella*) of Proteobacteria had increased relative abundance in the giant panda with bamboo consumption. The Bacteroidetes and Actinobacteria increased in adult giant panda with the relative abundance of 4%. The phyla Basidiomycota and Ascomycota were the dominant fungi in giant panda and increased abundance to 98% in adults. The genus *Trichosporon* of Basidiomycota and *Candida*, *Trimmatostroma*, Pleosporales and Montagnulaceae of Ascomycota were more common. However, the more abundant *Trichosporon* and *Candida* have been reported as pathogens in mammals, thus a hostile relationship might exist between fungi and the giant panda. There was no obvious dominant resident in fungal community and no positive correlation with the development of giant panda (Figure 4 and Supplementary Figures 1, 2).

According to the metagenomic analysis, a specific set of genes encoding endoglucanase (total value = 96) was collected from the above total non-redundant gene catalog and aligned to NR database for taxonomic annotation (Supplementary Table 3). The gut bacterial species possessing endoglucanase genes mainly included *Paenibacillus elgii*, *Lachnoclostridium phytofermentans*, *Clostridium longisporum*, and *Cellulosilyticum lentocellum* (Figure 4D). Among these species, *P. elgii*, *L. phytofermentans*, and *C. longisporum*, belonging to Firmicutes, were enriched in the giant panda on a bamboo diet, but all had a relative abundance <0.01% (Figure 4D). *Cellulosilyticum*, in the phylum Bacteroidetes, occurred momentarily in the dietary-shift period (Figures 4D,E), but was not detected in the sub-adult or adult giant panda; therefore, this genus was likely not able to colonize in a bamboo-fed giant panda. Members of the phylum Bacteroidetes are reported to degrade lignocellulose in herbivores at the genus level (Zhu et al., 2011; Yang et al., 2018; Guder and Krishna, 2019), such as the genus *Bacteroides* which has increased in the adult giant panda with a relative abundance of 2.25%. Low levels of cellulose-degrading fungi, such as *Aspergillus* (0.73%), *Penicillium* (1.67%), and *Trichoderma* (0.02%) were also detected (Carvalho et al., 2013; Gaurav et al., 2017; Figure 4F); however, these species were more abundant in cubs and even higher than in adults. In contrast, bacteria might be more important for the adaptation to bamboo diet of giant panda.

This study further found that the predominant species in Firmicutes and Proteobacteria, such as *Escherichia coli*, *Clostridium dakarense* and *Clostridium botulinum*, *Turicibacter sanguinis*, and *Terrisporobacter glycolicus* actively participated in soluble carbohydrate and amino acid metabolism; consistent with the analysis of digestive enzyme activity. In particular, according to the taxonomic annotation of genes involved in the amino acid metabolism, the species *E. coli*, *C. dakarense*, *C. botulinum, and T. sanguinis* contributed to the metabolism of 13 amino acid, such as alanine, aspartate, glutamate, arginine, proline, phenylalanine, and methionine, which were also the main composition of amino acid in bamboo.

In general, gut bacteria have been involved in assisting giant panda to adapt to eating bamboo by gradually enriching the cellulolytic community to digest bamboo cellulose. In addition, some dominant bacteria might cooperate with cellulolytic flora to mainly metabolize proteins and soluble carbohydrate in bamboo for giant panda.

## Discussion

### Delayed but increased expression of cellulose-degrading enzymes in the giant panda during adaptation to cellulose

The analysis showed that the changes in activities of endoglucanase and exoglucanase presented *U*-shaped profiles. Both cellulases increased in bamboo-fed giant pandas after dietary shift. However, compared with protease and amylase that returned abilities to previous levels in sub-adults or adults, cellulases activities in adults were still an order of magnitude worse than previously, showing delayed expression in giant panda with bamboo consumption.

When adapting to a new fibrous diet (e.g., bamboo, forage, or gluten), the activities of fiber-digesting enzymes in the guts of the giant panda, herbivores, and omnivores all show *U*-shaped enzyme activity profiles. Therefore, some adaptive process occurs in every mammal. The endoglucanase activity of the giant panda increased 10-fold in adults compared with when they first consumed bamboo, and that of herbivores increased 25-fold in adults (Hao et al., 2021). In addition, the gliadinase activity of omnivores increased two-fold in adults (Fernandez-Perez et al., 2020). The giant panda showed a more positively adaptive change to digesting bamboo cellulose than omnivores showed, but its ability was still far behind that of herbivores, and the activities of both endoglucanase and exoglucanase were always lower during the growth of the giant panda than in herbivores (Guo et al., 2018; Hao et al., 2021). There is no doubt that the lack of exoglucanase, which directly breaks down the tough structure of bamboo cellulose, may be one factor limiting the complete degradation of cellulose by giant panda.

With regard to cellulases activities being maintained at high levels in milk-fed cubs in this study, a similar phenomenon has also been detected in humans and many other mammals before weaning (e.g., gliadinase in humans and cellulases in calves and pandas). This is speculated to be related to early development and derived from maternal inheritance or early diet (Fernandez-Perez et al., 2020; Hao et al., 2021). The later cellulase properties, expression profiles and the structure of the gut flora may not be consistent with those in the milk-fed cubs in terms of the degradation of cellulose.

The properties (optimal pH) of endoglucanase in adults differ from those in cubs, which means they are different types of endoglucanase. Hao et al. (2021) have also shown that in calves, the nature of their cellulases might vary with weaning and the consumption of forage, and that the ruminal pH also changed with this dietary change. The optimal pH of endoglucanase tends to be weakly acidic in both herbivores and bamboo-fed giant pandas, close to the ruminal pH (Hao et al., 2021). Therefore, it was speculated that after the pandas began to consume bamboo, they tried to produce new cellulases that differed from those expressed in milk-fed cubs, in an attempt to adapt the intestinal environment to the degradation of new types of cellulose. This phenomenon is worthy of further study.

### Neither the abundance nor transcription of lignocellulose-decomposing genes in the gut microbial genome are sufficient for the giant panda to fully degrade bamboo cellulose

Gut microbial metagenomic analysis showed that the abundance of genes encoding endoglucanase (EC 3.2.1.4) was higher than for other lignocellulose-decomposing enzymes in the giant panda and increased with bamboo consumption. The two subtypes of GH5 (GH5_2 and GH5_4) and GH9 corresponding to endoglucanase (EC 3.2.1.4) have been detected in the microbial genome of bamboo-fed giant pandas, and GH9 is more helpful for hydrolyzing complex cellulose (Vita et al., 2019).

However, genes encoding GH9 (with a relative abundance of >0.01 among the GHs) were more abundant in herbivores, both with (e.g., cow) and without (e.g., elephant) rumens, than in the giant panda, and that more diverse suites of genes encoding GHs and carbohydrate-binding modules (CBMs) for lignocellulose degradation (such as GH44, GH45, GH48, GH74, CBM4, CBM6, CBM37, and CBM44 for cellulose degradation and GH10, GH26, GH28, GH53, CBM8, and CBM46 for hemicellulose degradation) were enriched and expressed during the growth and transition of herbivores to a plant-based diet (Supplementary Table 2; Shinkai et al., 2016; Ilmberger et al., 2017; Bohra et al., 2019).

Analysis of the transcriptome showed that only GH5 gene cluster maintained efficient transcription in bamboo-fed giant pandas for hydrolyzing cellulose. Compared with transcriptional silencing of GH9 in the giant panda, the transcriptional efficiency of GH9 was higher in herbivores than in the giant panda, accounting for 0.023–0.043 of GH transcripts (Supplementary Table 2; Dai et al., 2014). The transcriptional efficiency of GH5, encoding the most abundant cellulase GH, does not differ markedly between the giant panda and herbivores after they begin to consume plants (Shinkai et al., 2016). Moreover, the subtypes of GH5 found in the giant panda could potentially degrade cellulose and hemicellulose simultaneously (Glasgow et al., 2020; Pauchet et al., 2020).

However, compared with other herbivores, the giant panda lacks genes encoding more types of lignocellulose-decomposing enzymes for the hydrolysis of the complex celluloses in bamboo, in terms of their abundance and transcriptional efficiency. This largely explained the giant panda’s poor ability to adapt to the dietary shift and to fully degrade bamboo cellulose.

### A primary and amateur cellulose-degrading microbial community is formed to adaptively digest cellulose in the short carnivore-like gut of the giant panda

Consistent with the findings of Guo et al. (2020), the diversity of the gut flora decreased significantly in the giant panda after the dietary shift to that of sub-adults. Reduced diversity in the gut microbial population and gradually weakened complex interactions in the microbial community indicate adverse effects on the intestinal health of many animals (Tulstrup et al., 2018; Fassarella et al., 2021). They might also hinder the giant panda’s adaptation to high-cellulose diet, inducing the crisis observed in the giant panda. In addition, combined with the results of β-diversity analysis, the structure of the gut flora was immature and fragile at that time.

The dominant bacterial genera in adult pandas were still *Streptococcus*, *Escherichia–Shigella*, and *Clostridium*, which have long histories of survival in the human gut and those of other omnivores. These taxa have adequate strategies for obtaining energy (Berlemont and Martiny, 2013); probably including in high-cellulose niches (Williams et al., 2013; Song et al., 2017). Therefore, it was considered that the gut microorganisms of the giant panda are still predominantly in a struggle for survival, and have not yet become a specialized bamboo-degrading community. In this study, the predominant species preferred to metabolize the small amounts of protein and soluble carbohydrate in bamboo.

Some cellulose-degrading microbial populations were gradually enriched in the adult giant panda, but their abundance and activities were insufficient to support a high-cellulose bamboo diet. In herbivores, the ratio of Firmicutes to Bacteroidetes is an important index of the ability to digest plant material, and is on average 1:1 in different herbivorous animals. However, the proportion of Bacteroidetes in the giant panda is smaller (Güllert et al., 2016), which might explain the lack of a stable, strictly anaerobic gut environment, because its short intestine limits the length of stay for food (Megahed et al., 2019). In response to plant polysaccharides, the GHs in herbivores are expressed by a complex network of Bacteroidetes, Fibrobacteres, and Firmicutes (Jiang et al., 2011; Hao et al., 2021), whereas the cellulase GHs in the giant panda were mainly expressed by Firmicutes, and the community of cellulolytic microbes was still elementary and simple.

According to the systematic analysis and discussion of digestive enzymes, functional genes and microbial community, both the gut microbial structure and function have been developed to digest bamboo cellulose in the giant panda. However, their configurations were not able to fully degrade cellulose. Therefore, the giant panda cannot completely adapt to bamboo diet by only relying on cellulose-degrading flora.

### Potential strategies of the giant panda to maintain its normal growth and development without completely degrading bamboo cellulose

The studies reported above have indicated that the adaptive succession of the gut flora in the intestinal environment of the giant panda is still immature and amateur, and cannot fully convert cellulose into small-molecule nutrients like those in herbivores. It was anticipated that the microbial community had potential to produce new types of cellulases and continuously enhance its ability to digest bamboo. At present, the increased expression of endoglucanase might damage the bamboo cell walls and help release the intracellular degradable nutrients to the giant panda, which is likely to be one of the most important efforts by the giant panda to cope with bamboo diet. Some researchers have reported that the giant panda preferentially eats bamboo leaves where the main component is cellulose and there is accumulated crude protein, crude fat, sugar, and minerals (He, 2010; Wu et al., 2017).

To acquire even a small amount of energy and nutrient in bamboo, the giant panda must eat a large quantity (up to tens of kilograms per day). Because mucus is discharged by the giant panda after it foregoes a bamboo diet for a while, we inferred that a large amount of mucus generated in the intestine might protect the panda’s gastrointestinal tract, preventing damage by tough bamboo fiber. Whether the secretory mucus was actually an important strategy allowing its consumption of a lot of bamboo and/or has other functions, such as promoting the absorption of bamboo nutrients, required further research.

## Conclusion

From our results and the other research discussed above, several conclusions can be drawn:

1. During its dietary shift process, the giant panda gradually adapts to a bamboo diet, especially enhancing its ability to digest bamboo cellulose, but the activities of cellulases derived from gut microbes are still insufficient to convert cellulose to available nutrients.
2. A primary and amateur cellulolytic microbiota has been formed in giant panda, while the colonization of some efficient but anaerobic-preferred flora is limited by the short carnivore-like gut, resulting in the low abundance and transcription of cellulase genes and currently incomplete digestion of bamboo cellulose.
3. The giant panda appears most vulnerable during its dietary shift, especially when it first predominantly eats bamboo, because the intestinal flora structure is immature, so that the activities of digestive enzymes and the transcription of functional genes are lowest in this period.

## Data availability statement

The datasets presented in this study can be found in online repositories. The names of the repository/repositories and accession number(s) can be found below: https://www.ncbi.nlm.nih.gov/, PRJNA808648.

## Author contributions

MZ, AW, YZ, and SZ performed the material preparation. MZ, AW, XF, and JZ planned the experiments. MZ and XF performed the data collection and analysis. MZ wrote the first draft of the manuscript. LW revised the manuscript critically. YY, EP, and LW participated in the planning and coordination of the study. All authors commented on previous versions of the manuscript and read and approved the final manuscript.

## Conflict of interest

The authors declare that the research was conducted in the absence of any commercial or financial relationships that could be construed as a potential conflict of interest.

## Publisher’s note

All claims expressed in this article are solely those of the authors and do not necessarily represent those of their affiliated organizations, or those of the publisher, the editors and the reviewers. Any product that may be evaluated in this article, or claim that may be made by its manufacturer, is not guaranteed or endorsed by the publisher.
