# Supplementary material for: An amateur gut microbial configuration formed in giant panda for striving to digest cellulose in bamboo: Systematic evidence from intestinal digestive enzymes, functional genes and microbial structures
Source: Front Microbiol. 2022 Jul 26;13:926515. doi: 10.3389/fmicb.2022.926515 (PMC9363027; doi:10.3389/fmicb.2022.926515)
Supplement: Supplementary file 1 [file Data_Sheet_1.docx]

***Supplementary Material***

**An amateur gut microbial configuration for giant panda** **striving to digest cellulose in bamboo: systematic evidence from intestinal digestive enzymes, functional genes and microbial structures**

**Includes:**

**Supplementary Methods**

**Supplementary Tables**

**Supplementary Figures**

- **Supplementary Methods**
- **DNA extraction**

The DNA was extracted from the filter membranes with the PowerSoil® DNA Isolation Kit (Mo Bio Laboratories Inc., Carlsbad, CA, USA), according to the protocol of manufacturer. Each 0.5 g stool sample was inserted into the “Power Bead Tubes” and manually mixed. The C1 solution (dissolved at 60 °C) was added, and the sample was vortexed for 10 min and centrifuged for 1 min at 13000 g. According to the protocol, the C2 and C3 solutions were added to the collected supernatant in turn, and the mixture incubated at 4 °C for 5 min with each addition. The collected supernatant was mixed with C4 solution and loaded onto a “Spain Filter”. The DNA stayed bound to the filter membrane, and was washed with C5 solution, and then eluted and collected with ddH2O. The DNA concentration and quality were evaluated with a NanoDrop™ 2000 spectrophotometer (NanoDrop, Wilmington, DE, USA).

- **16S and ITS-1 sequence amplification**

The bacterial DNA was amplified with the primers 338F (5’-ACTCCTACGGGAGGCAGCAG-3’) and 806R (5’-GGACTACHVGGGTWTCTAAT-3’). Unique eight-base-sequence barcodes were attached to each sample. The PCRs were performed in triplicate in 20 μL reaction mixtures containing 4 μL of 5 × FastPfu Buffer, 2 μL of 2.5 mM dNTPs, 0.4 μL of each primer (10 μM), 0.4 μL of FastPfu Polymerase, 10 ng of template DNA, and ddH2O to the final volume. The amplicons were extracted from 2% agarose gels and purified with the AxyPrep DNA Gel Extraction Kit (Axygen Biosciences, Union City, CA, USA), according to the manufacturer’s instructions. The amplicons were quantified with QuantiFluor™-ST (Promega Corporation, Madison, WI, USA). Equimolar amounts of the purified amplicons were pooled and paired-end sequenced on an Illumina MiSeq 300 platform, with standard protocols. Fungi were amplified with the primers ITS1F (5’-CTTGGTCATTTAGAGGAAGTAA-3’) and ITS2 (2043R) (5’-GCTGCGTTCTTCATCGATGC-3’). The PCRs were performed in triplicate in 20 μL reaction mixtures containing 2 μL of 10 × buffer, 2 μL of 2.5 mM dNTPs, 0.2 μL of rTaq polymerase, 0.4 μL of each primer (10 μM), 0.4 μL of FastPfu Polymerase, 10 ng of template DNA, and ddH2O to the final volume.

- **Quality for 16S and ITS-1 sequencing**

The raw fastq files of 16 S and ITS rDNA were demultiplexed and quality-filtered by fastp version 0.19.6 and merged by FLASH, according to the following criteria: (i) the 300 bp reads were truncated at any site receiving an average quality score of <20 over a 50 bp sliding window, and the truncated reads shorter than 50 bp were discarded, reads containing ambiguous characters were also discarded; (ii) only overlapping sequences longer than 10 bp were assembled according to their overlapped sequence. The maximum mismatch ratio of overlap region was 0.2. Reads that could not be assembled were discarded; (iii) samples were distinguished according to the barcode and primers, and the sequence direction was adjusted, exact barcode matching, two-nucleotide mismatch in primer matching.

- **Construction of paired-end library and DNA sequence qualification**

The quality of the extracted fecal DNA of giant panda was checked on 1% agarose gel, and the DNA was then fragmented to an average size of about 400 bp using the Covaris M220 Focused ultrasonicator (Gene Company Ltd., China) to construct a paired-end library. Adapters containing the full complement of sequencing primer hybridization sites were ligated to the blunt-ended fragments. Paired-end sequencing was performed on the Illumina NovaSeq platform (Illumina Inc., San Diego, CA, United States) at Majorbio Bio-Pharm Technology Co., Ltd. (Shanghai, China). The data were analyzed on the free online platform Majorbio Cloud Platform. The paired-end Illumina reads were qualified with trimmed of adaptors, and low-quality reads (length < 50 bp; with a quality value < 20; or containing N bases) were removed with Transcriptomic Sequence Analysisfastp (version 0.20.0). The metagenomic data were assembled with MEGAHIT (version 1.1.2), which makes use of succinct de Bruijn graphs. Contigs with a length of 300 bp were selected as the final results for assembly, and the contigs were then used for gene prediction and annotation.

- **Annotation of Metagenomic sequences**

Representative sequences from the NR gene catalog were aligned to those in NCBI NR database for taxonomic annotation using Diamond (version 0.8.35) (according to best-hit method with an e value cutoff of 1e-5), against the eggNOG database for cluster of orthologous groups (COG) of proteins annotation, using Diamond (version 0.8.35) (with an e-value cutoff of 1e-5), and against the Kyoto Encyclopedia of Genes and Genomes (KEGG) database for KEGG annotation, using Diamond (version 0.8.35)10 (with an e-value cutoff of 1e-5). The carbohydrate-active enzymes were annotated with hmmscan11 against the CAZy database, with an e-value cutoff of 1e-5.

- **Construction of paired-end library and cDNA sequence qualification**

The cDNA library was constructed using the TruSeq Stranded mRNA LT Sample Prep Kit (Illumina Inc., San Diego, CA, United States). Firstly, the RNA was fragmented by divalent cation under high-temperature condition and then the mRNA was reversely transcribed to cDNA (synthesis of the first chain: fragmented mRNA with First Strand Synthesis Act D Mix and Superscript III (9:1) at 25℃, 10 min→42℃, 50 min→85℃, 15→4℃, ∞; synthesis of the second chain: mixed with Second Strand Marking Master Mix and End Repair Control at 16℃, 1h) and purified with BECKMAN AMPure XP beads for library construction. The filtered (with adding tail A to 3` and adding specific labelled linker to hybridize to Flow cell) and purified (with BECKMAN AMPure XP beads) sequences were qualified on the Agilent 2100 Bioanalyzer (Agilent Technologies Inc., Beijing, China) with Agilent High Sensitivity DNA Kit. The qualified library only had a single peak without joints and then was quantified on the Promega QuantiFluormeter (Promega Corporation, Beijing, China) with Quant iT PicoGreen dsDNA Assay Kit, satisfying the qualified standard >2nM.

- **Supplementary Tables**

**Table S1 The basic information of giant pandas.**

| **Sample number** | **Date of birth** | **Developmental stage for collection** | **Gender** | **Habitat** |
| --- | --- | --- | --- | --- |
| **S1** | **2017.6** | **Cub; Dietary-shift** | **♀** | **Chongqing** |
| **S2** | **2017.6** | **Cub; Dietary-shift** | **♂** | **Chongqing** |
| **S3** | **2017.6** | **Cub; Dietary-shift** | **♀** | **Chongqing** |
| **S4** | **2017.6** | **Cub; Dietary-shift** | **♂** | **Chongqing** |
| **S5** | **2016.4** | **Cub** | **♂** | **Shanghai** |
| **S6** | **2016.4** | **Cub** | **♀** | **Shanghai** |
| **S7** | **2016.7** | **Cub** | **♀** | **Sichuan** |
| **S8** | **2016.8** | **Cub** | **♀** | **Sichuan** |
| **S9** | **2016.7** | **Sub-adult** | **♀** | **Chongqing** |
| **S10** | **2016.7** | **Sub-adult** | **♂** | **Chongqing** |
| **S11** | **2013.8** | **Sub-adult** | **♂** | **Shanghai** |
| **S12** | **2013.8** | **Sub-adult** | **♂** | **Shanghai** |
| **S13** | **2013.8** | **Sub-adult** | **♂** | **Sichuan** |
| **S14** | **2013.8** | **Sub-adult** | **♂** | **Sichuan** |
| **S15** | **2000.8** | **Sub-adult** | **♂** | **Chongqing** |
| **S16** | **2005.8** | **Adult** | **♂** | **Chongqing** |
| **S17** | **2004.8** | **Adult** | **♂** | **Shanghai** |
| **S18** | **1998.7** | **Adult** | **♀** | **Shanghai** |
| **S19** | **1999.8** | **Adult** | **♀** | **Shanghai** |
| **S20** | **1999.9** | **Adult** | **♀** | **Sichuan** |
| **S21** | **2004.8** | **Adult** | **♂** | **Sichuan** |
| **S22** | **1995.8** | **Adult** | **♀** | **Sichuan** |

**Table S2 The relative gene abundance (gene counts/total GHs counts) of cellulase-GHs in giant panda and other herbivores.**

| **Metagenomic analysis** | | | | **Transcriptomic analysis** | | | |
| --- | --- | --- | --- | --- | --- | --- | --- |
|  | **giant panda (this study)** | **cow (Shinkai et al., 2016)** | **elephant (ilmberger et al., 2014)** |  | **giant panda (this study)** | **cow (Dai et al., 2015)** | **elephant**  **(Güllert et al., 2016)** |
| **GHs/toltal gene counts** | **1.6%** | **——** | **1.0%** | **GHs/toltal gene counts** | **——** | **0~1.0%** | **——** |
| **GH5/GHs gene counts** | **0.56%** | **0.61~5.5%** | **4.7%** | **GH5/GHs gene counts** | **0.94%** | **1.9~2.7%** | **6.2%** |
| **GH9/GHs gene counts** | **0.19%** | **0.55~3.0%** | **1.10%** | **GH9/GHs gene counts** | **——** | **2.25~4.3%** | **4.0%** |
| **GH44/GHs gene counts** | **——** | **——** | **0.063%** | **GH44/GHs gene counts** | **——** | **0.71~1.2%** | **0.25%** |
| **GH45/GHs gene counts** | **——** | **0~0.41%** | **0.063%** | **GH45/GHs gene counts** | **——** | **1.7~1.9%** | **——** |

- **Supplementary Figures**

**Figure S1 The phylogenetic tree and relative abundance of dominant intestinal bacteria in the giant panda at the genus level (top 30).**


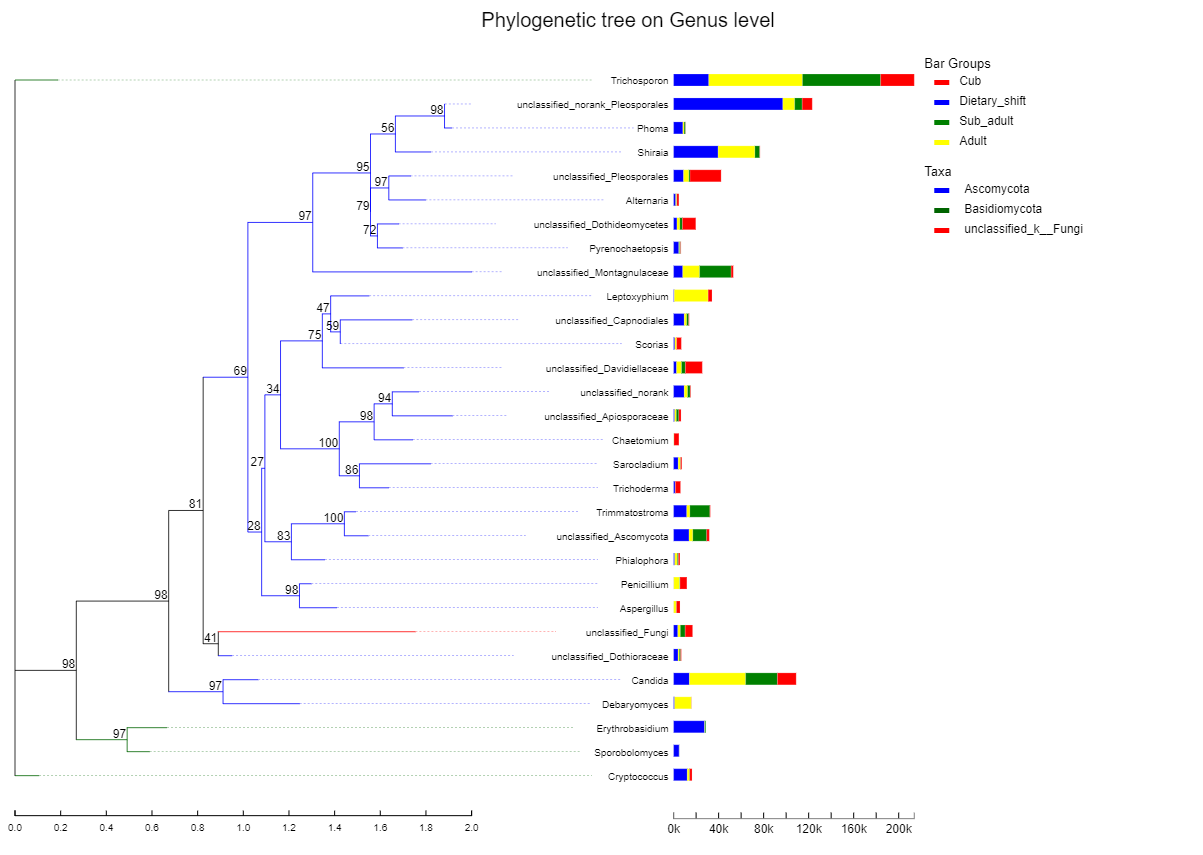

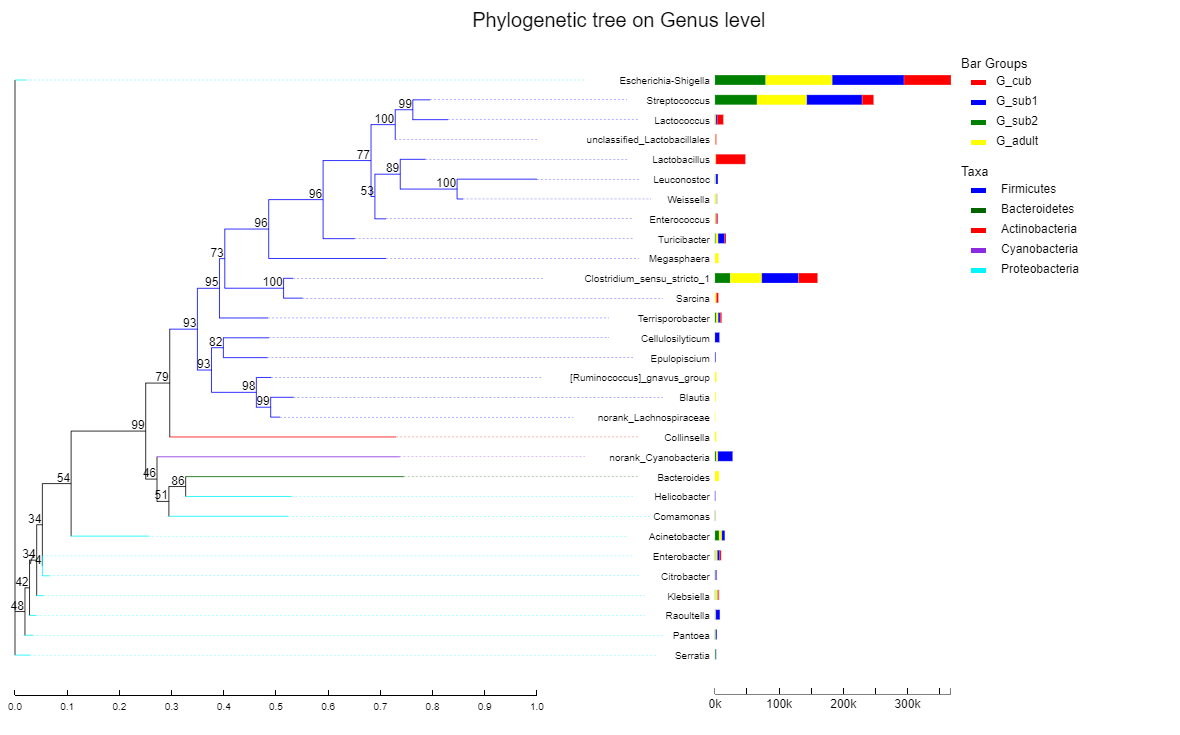

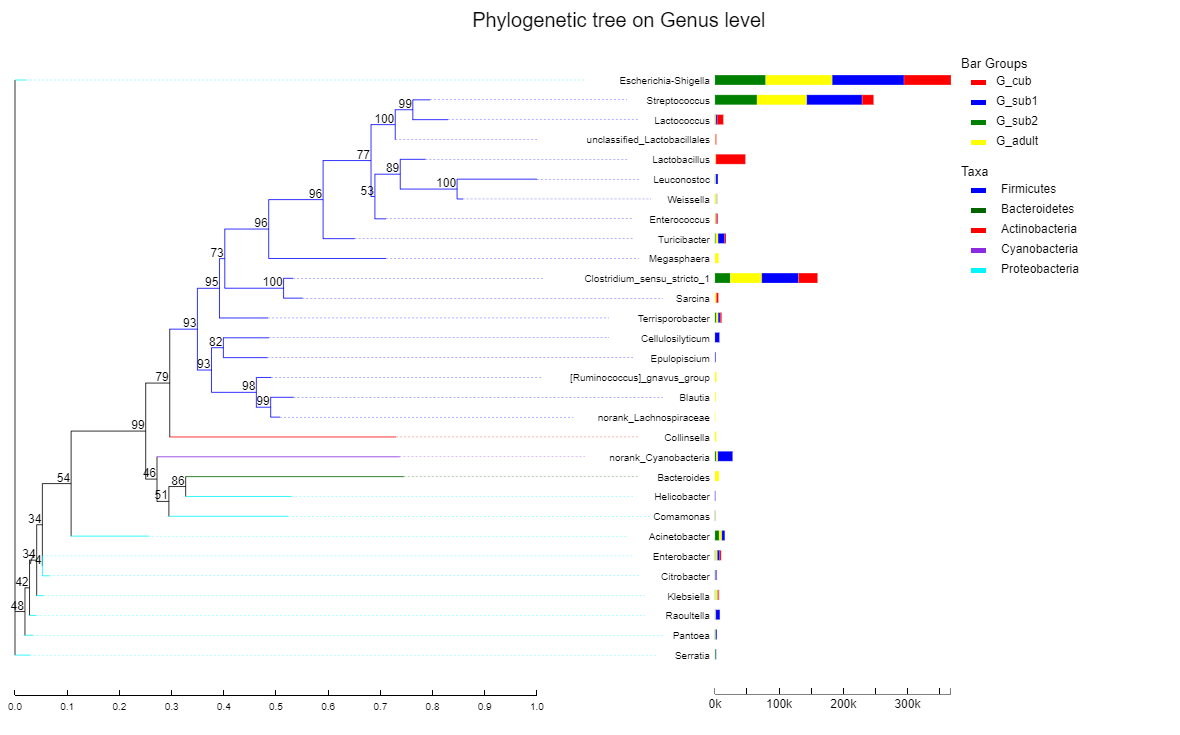


The left half showed the phylogenetic tree on genus level of the dominant bacteria in the giant panda constructed with Maximum Likelihood and the phyla they belong to, mainly Firmicutes (blue), Bacteroidetes (green), Actinobacteria (red), Cyanobacteria (purple) and Proteobacteria (azure). The right half showed the relative abundance of the dominant bacteria in the giant pandas at different developmental stages, including cub (blue), dietary-shift stage (green), sub-adult (red), adult (yellow).

**
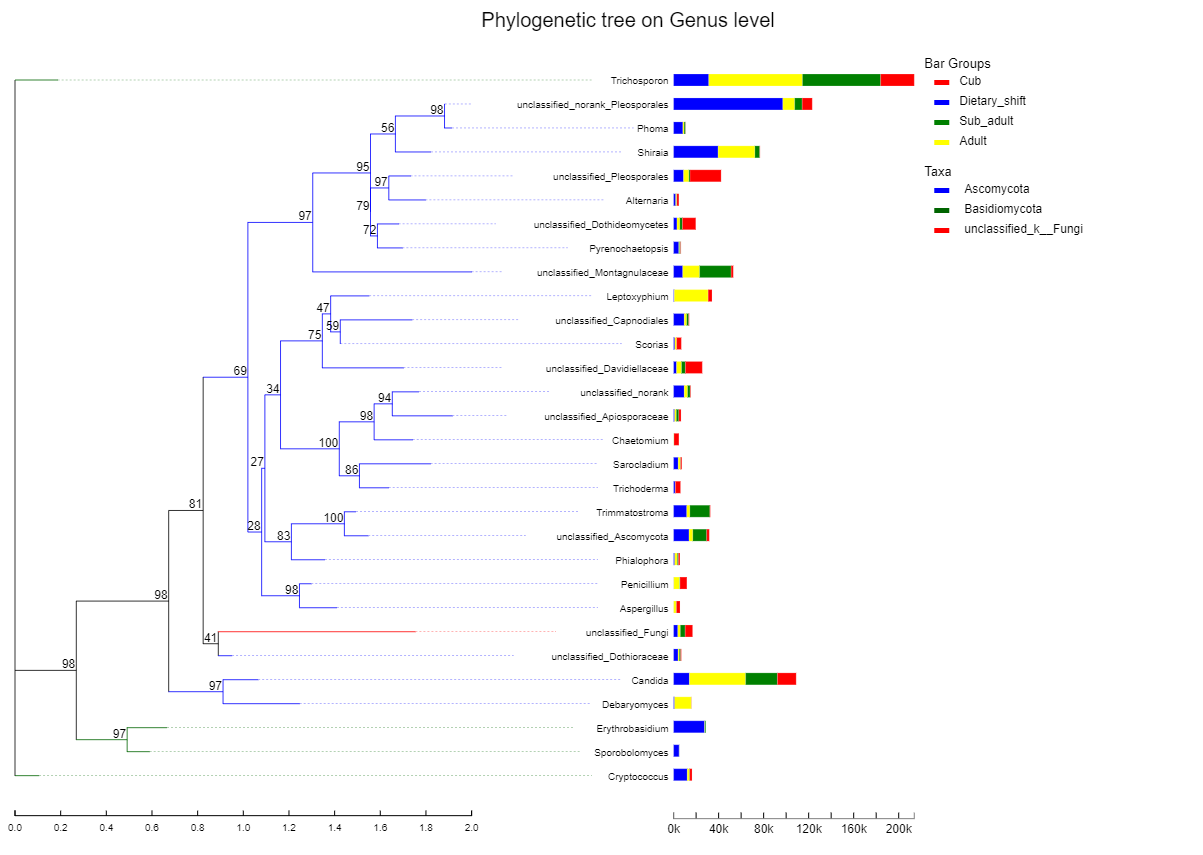
**

**Figure S2 The phylogenetic tree and relative abundance of dominant intestinal fungi in the giant panda at the genus level (top 30).**

The left half showed the phylogenetic tree on genus level of the dominant fungi in the giant panda constructed with Maximum Likelihood and the phyla they belong to, mainly Ascomycota (blue), Basidiomycota (green) and unclassified_k_Fungi (red). The right half showed the relative abundance of the dominant fungi in the giant pandas at different developmental stages, including cub (blue), dietary-shift stage (green), sub-adult (red), adult (yellow).
